# Supplementary material for: Integrative analysis reveals extensive interactions among C2H2 zinc finger proteins at chromatin loop anchors
Source: Nucleic Acids Res. 2026 Oct 19;54(19):gkag1004. doi: 10.1093/nar/gkag1004 (PMC13678222; doi:10.1093/nar/gkag1004)
Supplement: Supplementary file 1 [file gkag1004_supplemental_files.zip › Supplemental_figures.pdf]

Supplemental Figure S1

A

Statistic being reported	Replica 1	Replica 2
Total PETs	529,138,548	339,527,099
Valid PETs after linker filtering	382,075,028	234,529,648
Uniquely Mapped same-linker PETs	316,591,088	197,367,605
Merging same same-linker PETs	72,994,659	41,220,546
Merging similar same-linker PETs	61,475,616	35,913,991
Self-ligation PETs	28,182,358	14,690,011
Inter-ligation PETs	12,909,604	7,532,434
Other PETs with short distance	20,383,654	13,691,546
Peaks from self-ligation PETs	5,815	4,439
Signficant long range interactions	729,107	624,133
Signficant interchromosomal long range interactions	109,733	78,528
Signficant intrachromosomal long range interactions	619,374	545,605
Reproducible singificant long range interactions	63,511	
Significant interchromosomal long range interactions that are reproducible	177	
Significant intrachromosomal long range interactions that are reproducible	63,334	

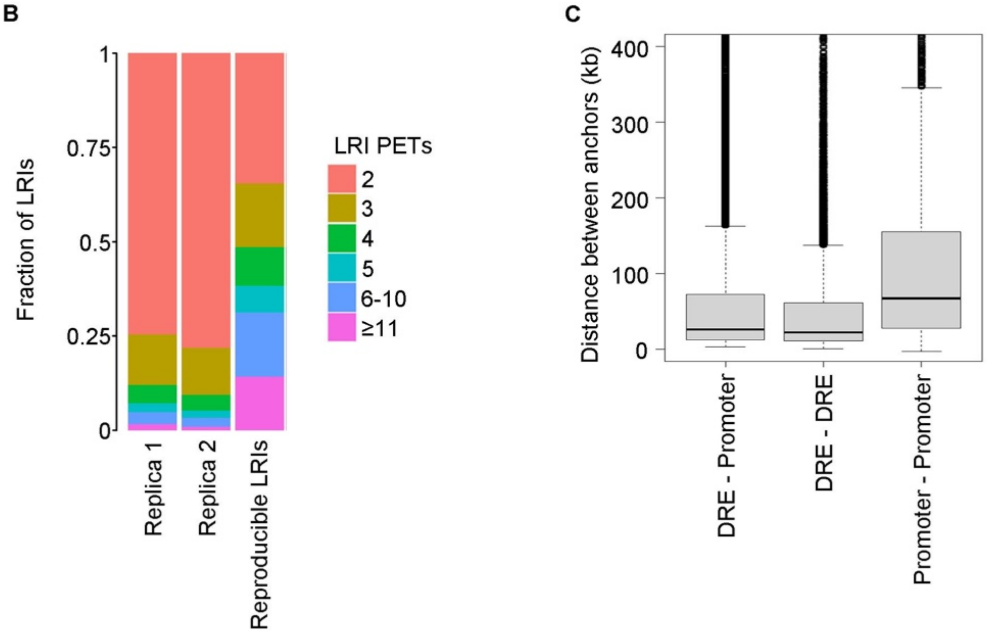

Supplemental Figure S1. Analysis of RNAP II ChIA-PET data.
(A) Table listing some statistics on the analysis of the two RNAP II ChIA-PET datasets in HEK293 cells.
(B) Barplot showing the fraction of LRIs that are linked by the indicated number of PETs for the reproducible LRIs, as well as the LRIs that were identified from the two individual RNAP II ChIA-PET replicate experiments.
(C) Boxplots showing the distribution of the distance between two different anchors for the indicated LRI types

Supplemental Figure 2

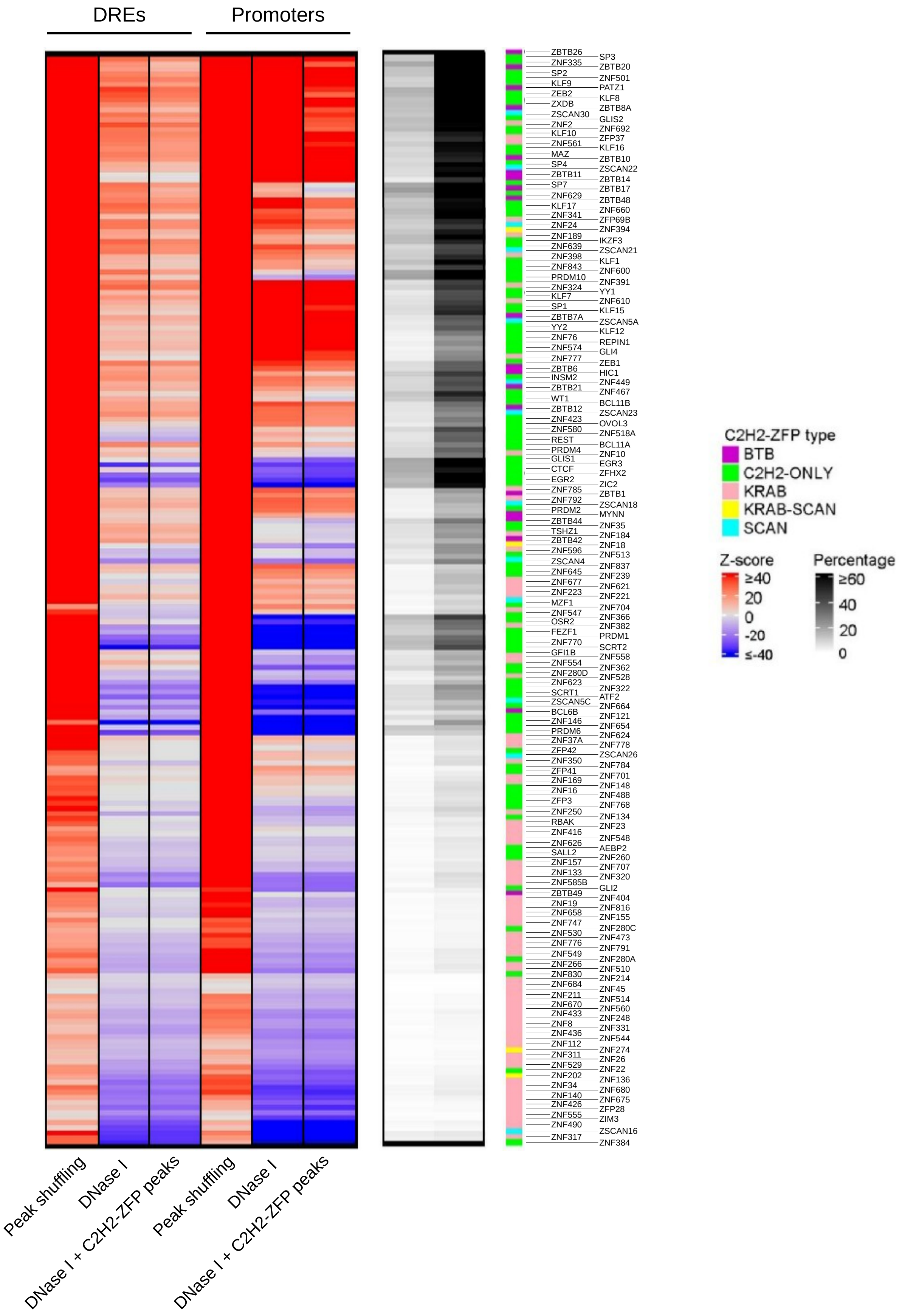

Supplemental Figure S2. Overlap of C2H2-ZFPs DNA-binding sites with LRI anchors. Heatmaps showing Z-scores representing the enrichment of C2H2-ZFP DNA-binding sites at LRI anchors that overlap DREs or promoters (left), together with the percentage of anchors bound by each C2H2-ZFP (right). Enrichment was assessed by comparing the observed overlap between C2H2-ZFP ChIP-seq peaks and LRI anchors to three null models: (i) chromosome- and size-matched random genomic locations generated for each C2H2-ZFP ChIP-seq dataset ("Random matched genomic intervals"), (ii) HEK293 DNase I hypersensitive sites ("DNase I"), and (iii) HEK293 DNase I hypersensitive sites together with the ChIP-seq peaks of all 216 analyzed C2H2-ZFPs ("DNase I + C2H2-ZFP peaks"). Positive Z-scores indicate enrichment of C2H2-ZFP binding at LRI anchors relative to the corresponding background model. Only statistically significant Z-scores are displayed. Note: This figure is the enlarged version of the main Figure 1B with gene names labeled.

Supplemental Figure S3

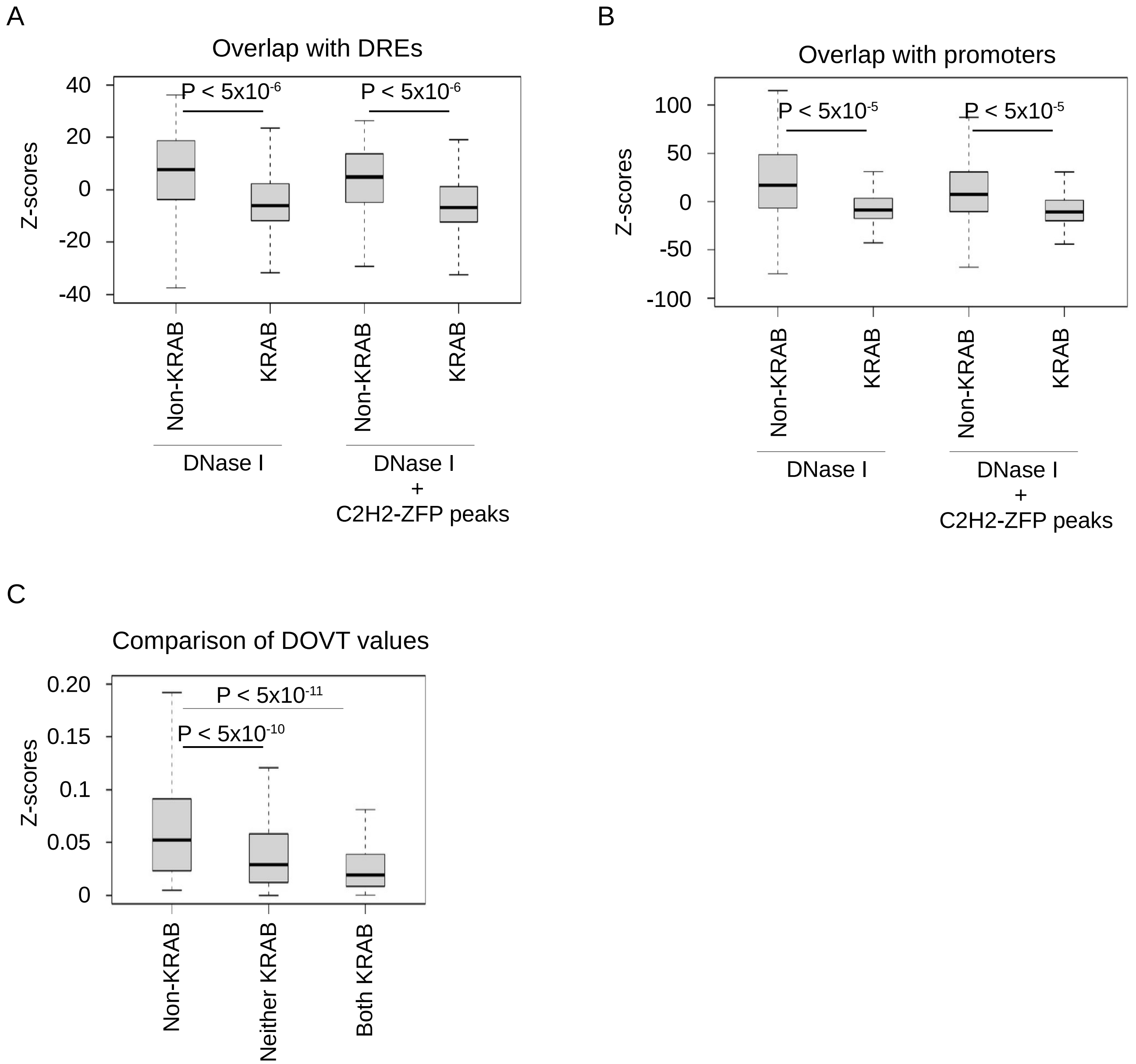

Supplementary Figure S3. KRAB-containing C2H2-ZFPs are depleted from RNAP II-associated chromatin loop anchors and exhibit reduced overlap with long-range chromatin interactions.

(A) Comparison of Z-scores for the overlap of KRAB and non-KRAB C2H2-ZFPs with distal regulatory element (DRE) anchors of RNAP II-associated long-range interactions (LRIs). Overlaps were evaluated using the DNase I and DNase I + C2H2-ZFP peaks background models described in Figure 1B. Non-KRAB C2H2-ZFPs exhibited significantly higher enrichment at DRE anchors than KRAB-containing C2H2-ZFPs (Mann–Whitney test).

(B) Comparison of Z-scores for the overlap of KRAB and non-KRAB C2H2-ZFPs with promoter anchors of RNAP II-associated LRIs using the same background models as in (A). Non-KRAB C2H2-ZFPs showed significantly greater enrichment at promoter anchors than KRAB-containing C2H2-ZFPs (Mann–Whitney test).

(C) Comparison of DOVT values for interacting C2H2-ZFP pairs containing no KRAB proteins (Non-KRAB), one KRAB-containing protein (Either KRAB), or two KRAB-containing proteins (Both KRAB). C2H2-ZFP pairs containing one or two KRAB proteins exhibited significantly lower DOVT values than pairs lacking KRAB proteins (Mann–Whitney test), indicating reduced association with RNAP II-associated long-range chromatin interactions.

Supplemental Figure S4

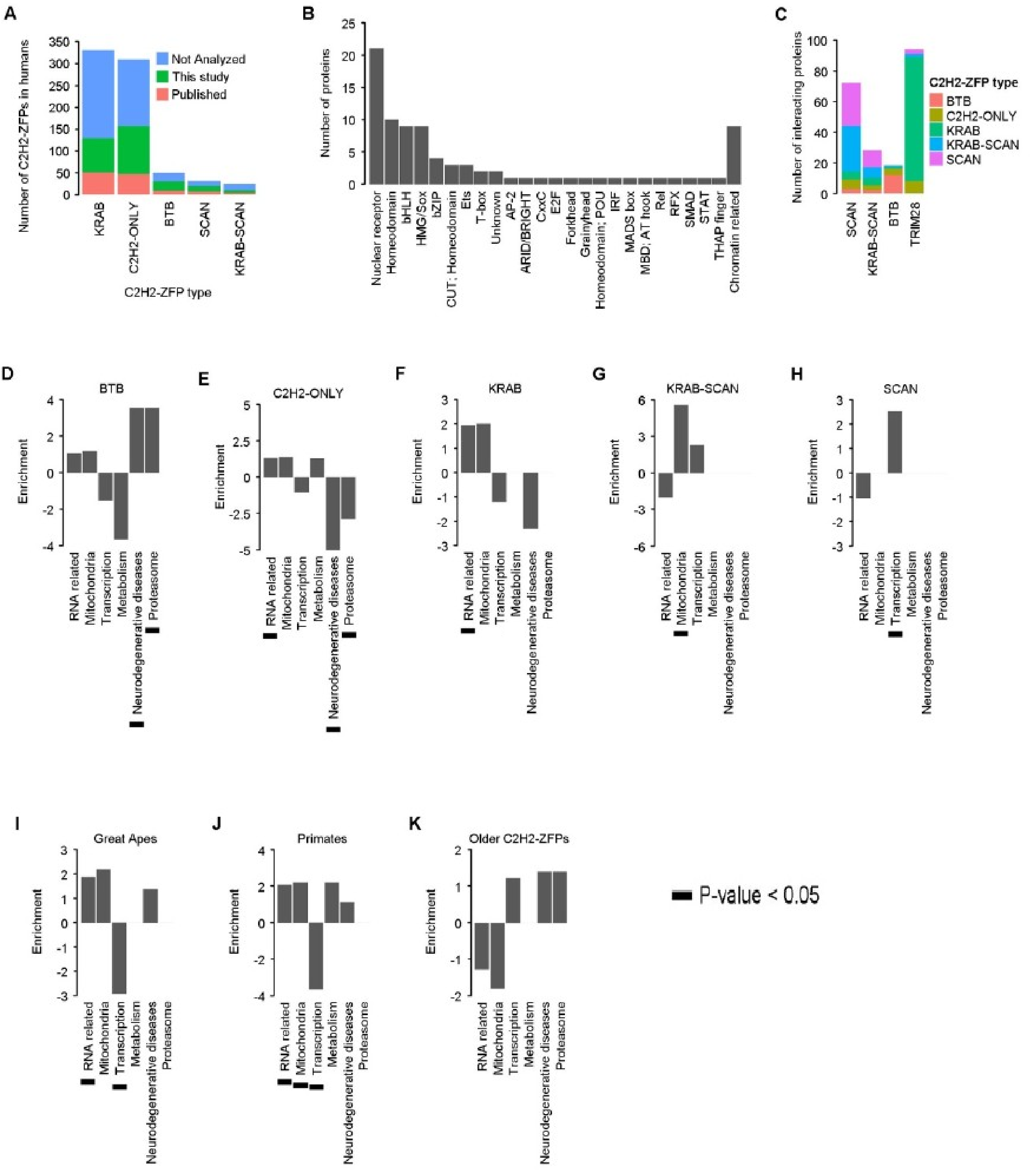

Supplemental Figure S4. PPI network of C2H2-ZFPs uncovers diverse interactions involved in multiple biological processes.

(A) Summary of C2H2-ZFP subfamilies that were characterized by AP/MS.

(B) Barplot showing the subfamilies to which the TFs used as background in the SAINT analysis belong. The 9 non-TF proteins used in the background were classified as “Chromatin related”.

(C) Barplot showing that AP/MS for C2H2-ZFPs captures many PPIs with other C2H2-ZFPs that are expected for C2H2-ZFPs based on auxiliary domains. For TRIM28 as a prey protein, or for baits containing a KRAB, SCAN or BTB domain, the types of interacting C2H2-ZFPs are classified according to their auxiliary domains.

(D-K) Barplots showing the enrichment of selected functional terms among PPI preys of (D) BTB-C2H2-ZFPs, (E) C2H2-ONLY-ZFPs, (F) KRAB-C2H2-ZFPs, (G) KRAB-SCAN-C2H2-ZFPs, (H) SCAN-C2H2-ZFPs, (I,J) C2H2-ZFPs found predominantly in (I) Great Apes or (J) Primates and (K) older C2H2-ZFPs. The p-values were calculated using the hypergeometric test, and they were corrected using the Benjamini-Hochberg method.

Supplemental Figure S5

A

SAINT score	Good preys	Possible peptide misidentifications	Possible carryovers	Percentage of filtered preys
1	101	14	15	22.3
0.99	51	11	1	19.1
0.98	10	1	0	9.1
0.97	52	22	4	33.3
0.96	11	2	0	15.4
0.95	4	0	1	20.0
0.94	41	41	0	50.0
0.93	9	1	0	10.0
Total	279	92	21	28.8

B

	C2H2-ONLY (35)	KRAB (43)	KRAB-SCAN (6)	SCAN (12)	BTB (8)
C2H2-ONLY (27)	36	11	2	2	3
KRAB (41)	7	117	0	2	1
KRAB-SCAN (8)	3	8	7	30	1
SCAN (6)	0	1	11	25	0
BTB (10)	3	0	0	1	8

C

	C2H2-ONLY (61)	KRAB (89)	KRAB-SCAN (13)	SCAN (13)	BTB (26)
C2H2-ONLY (18)	254	368	69	50	98
KRAB (20)	249	334	70	57	96
KRAB-SCAN (1)	16	15	8	8	6
SCAN (1)	5	4	4	4	0
BTB (5)	56	49	11	7	27

D

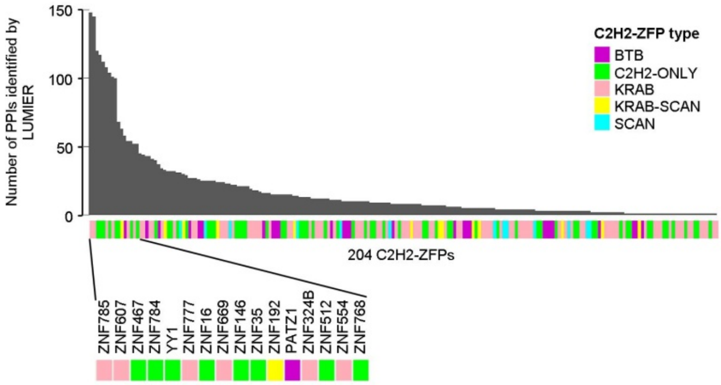

E

Bait proteins	282
Prey proteins	54
Proteins tested as both bait and prey	52
Interactions tested by LUMIER	13850
Interactions confirmed by LUMIER	1732
AP-MS PPIs tested by LUMIER	74
AP-MS PPIs confirmed by LUMIER	21
AP-MS PPIs expected to be confirmed by chance	9.25
P-value	<0.0005

F

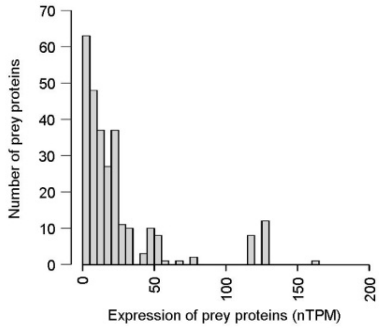

G

TPM cutoff for prey proteins	0	1	5
Bait proteins	345	345	345
Prey proteins	751	609	401
Interactions tested by AP/MS	199410	150420	78660
Interactions confirmed by AP/MS	278	278	278
LUMIER interactions tested by AP/MS	1656	1656	1656
LUMIER interactions confirmed by AP/MS	21	21	21
LUMIER interactions expected to be confirmed	2.31	3.06	5.85
p-value	$<5x10^{-14}$	$<5x10^{-11}$	$<5x10^{-7}$

Supplemental Figure S5. C2H2-ZFPs interact with each other extensively.

(A) Table summarizing the filtering of PPIs due to prey peptides that could have been misidentified by mass spectrometry at different SAINT cutoffs.

(B) Summary of the number of PPIs among C2H2-ZFPs from different subfamilies based on the AP/MS network from Fig. 3C. The numbers inside brackets in the column headings represent the number of unique bait C2H2-ZFPs displaying PPIs. The numbers inside brackets in the row labels represent the number of unique prey C2H2-ZFPs identified as interactors.

(C) Summary of the number of PPIs among C2H2-ZFPs from different subfamilies based on LUMIER PPIs from Fig. 3D. The numbers inside brackets represent the same types of C2H2-ZFPs as in panel B.

(D) Barplot displaying the number of interactions that LUMIER identified for each C2H2-ZFP that was tested.

(E) Table with statistics assessing whether LUMIER confirms more AP/MS C2H2-ZFP PPIs than expected by chance. The p-value was calculated using the hypergeometric test.

(F) Histogram displaying the distribution of expression levels of prey C2H2-ZFP proteins in HEK293 cells that appear in the C2H2-ZFP interacting pairs that were identified by AP/MS. C2H2-ZFPs that are members of pairs that could have been misidentified because of mass spectrometry artifacts are not included.

(G) Table with statistics assessing whether AP/MS confirms more LUMIER C2H2-ZFP PPIs than expected by chance. Calculations were done by using different TPM values as a cutoff for the expression of prey C2H2-ZFPs in HEK293 cells. The p-values were calculated using the hypergeometric test.

Supplemental Figure S6

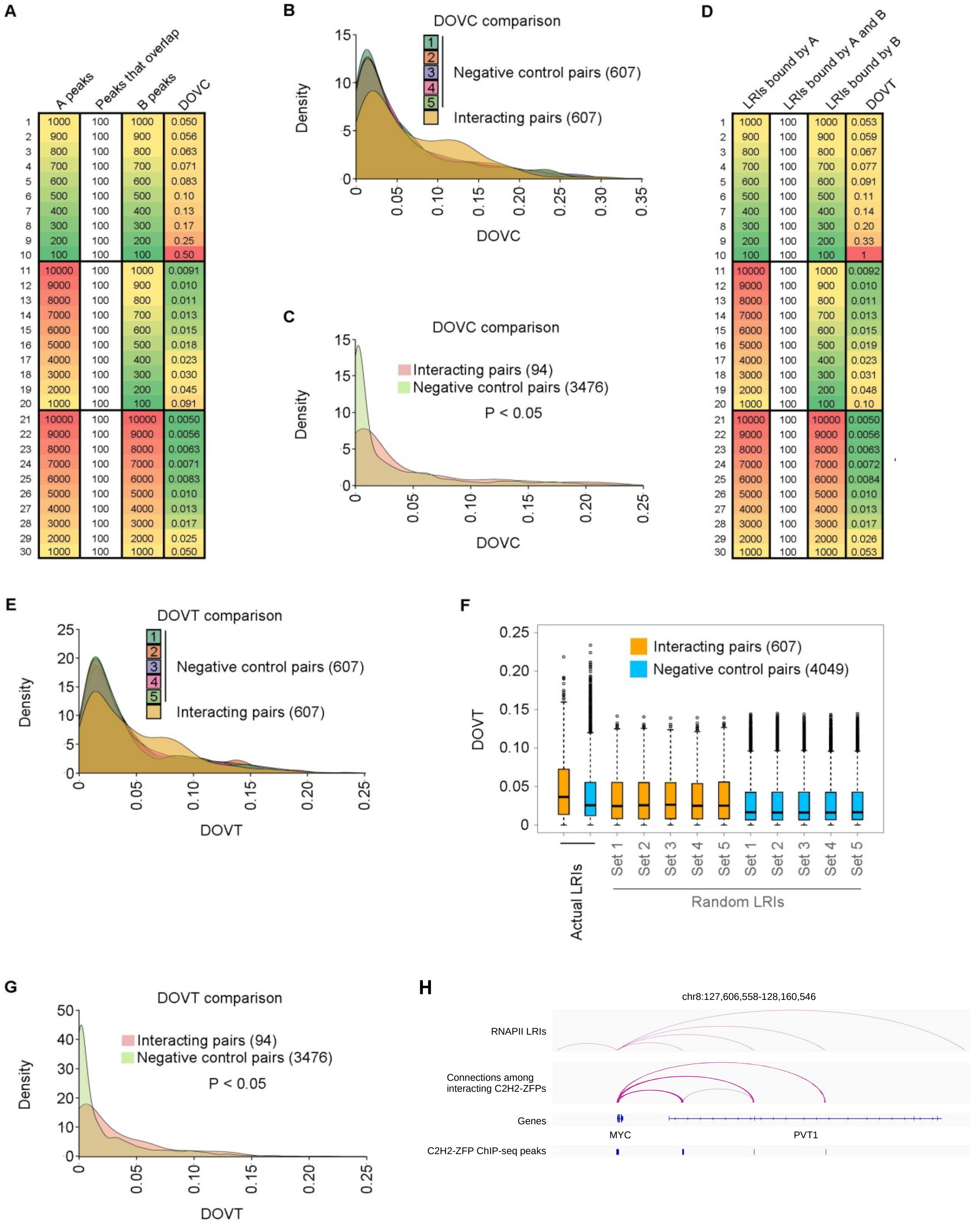

Supplemental Figure S6. Interacting C2H2-ZFP proteins co-localize significantly at the same as well as at the opposite ends of LRIs.

(A) Table showing hypothetical examples of how the DOVC value could change for C2H2-ZFPs when they exhibit different degrees of overlap in ChIP-seq peaks.

(B) Density plots comparing distributions of DOVC values for interacting C2H2-ZFP pairs from LUMIER to negative controls. The DOVC values were calculated using only DNA-binding sites that overlap with LRI anchors. Negative controls consist of five sets of 607 randomly chosen pairwise C2H2-ZFP combinations that were not found to interact by LUMIER. p-values comparing interacting pairs to the five sets of negative controls were calculated using the Mann-Whitney test, corrected using the Benjamini-Hochberg method, and they are listed in Supplemental Table S12A.

(C) Density plots comparing distributions of DOVC values for interacting C2H2-ZFP pairs from AP/MS to negative controls. The DOVC values were calculated using only DNA-binding sites that overlap with LRI anchors. p-values were calculated using the Mann-Whitney test. Negative controls consist of all pairwise C2H2-ZFP combinations that were not found to interact by AP/MS.

(D) Table showing hypothetical examples of how the DOVT value could change for C2H2-ZFPs when they exhibit different degrees of overlap in DNA-binding sites at LRIs.

(E) Density plots comparing distributions of DOVT values for interacting C2H2-ZFP pairs to negative controls. Negative controls consist of five sets of 607 randomly chosen pairwise C2H2-ZFP combinations that were not found to interact by LUMIER. p-values comparing interacting pairs to the five sets of negative controls were calculated using the Mann-Whitney test, corrected using the Benjamini-Hochberg method, and they are listed in Supplemental Table S12B.

(F) Boxplots comparing DOVT values of all interacting C2H2-ZFPs pairs that were identified by LUMIER to various negative controls that include 1) DOVT values that were calculated for all the pairwise combinations of C2H2-ZFPs that were not found to interact by LUMIER using the DRE-promoter LRIs (Actual LRIs), 2) DOVT values that were calculated for all interacting C2H2-ZFP pairs using five sets of shuffled DRE-promoter LRIs, and 3) DOVT values that were calculated for all the pairwise combinations of C2H2-ZFPs that were not found to interact by LUMIER using the same five sets of shuffled DRE-promoter LRIs that were used for the interacting pairs. p-values comparing interacting pairs to negative controls were calculated using the Mann-Whitney test, corrected using the Benjamini-Hochberg method, and they are listed in Supplemental Table S12C.

(G) Density plots comparing distributions of DOVT values for interacting C2H2-ZFP pairs from AP/MS to negative controls at LRIs anchors. p-values were calculated using the Mann-Whitney test. Negative controls consist of all pairwise C2H2-ZFP combinations that were not found to interact by AP/MS.

(H) Genome browser snapshot of selected locus showing the potential connections among interacting C2H2-ZFPs. Top panel: RNAPII LRIs from ChIA-PET dataset. Second panel (from the top): Connections among interacting C2H2-ZFPs that bind at the anchors of the RNAPII LRIs. Third panel: Locations and names of genes in the depicted locus. Fourth panel: Location of the ChIP-seq summits for the interacting C2H2-ZFPs in the second panel.

Supplemental Figure S7

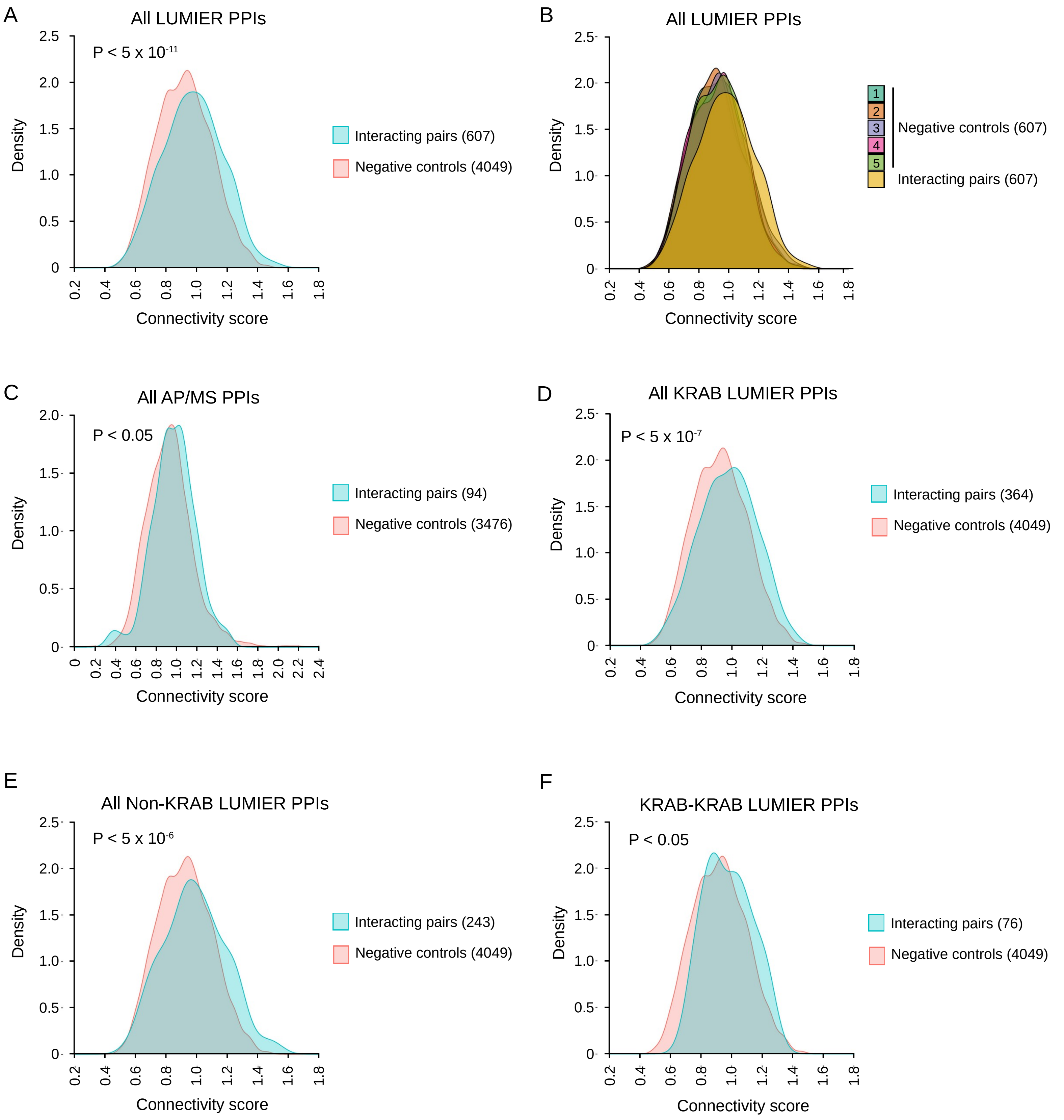

Supplemental Figure S7: Interacting C2H2-ZFP pairs preferentially occupy highly connected chromatin regions in Hi-C datasets from HEK293 cells.

(A) Density plot comparing the average Hi-C connectivity scores of chromatin bin pairs occupied by interacting LUMIER C2H2-ZFP pairs (607 pairs) and negative control C2H2-ZFP pairs (4,049 pairs) that were not detected to interact. Interacting pairs exhibited significantly higher connectivity scores than negative controls (Mann–Whitney test).

(B) Density plot comparing the average Hi-C connectivity scores of the 607 interacting LUMIER C2H2-ZFP pairs with five independently selected, equal-sized random subsets of negative control pairs (607 pairs each). In all five comparisons, interacting C2H2-ZFP pairs exhibited significantly higher connectivity scores than the corresponding negative control set (Mann–Whitney test; Set 1, $P = 3.16 \times 10^{-7}$; Set 2, $P = 3.16 \times 10^{-7}$; Set 3, $P = 5.98 \times 10^{-6}$; Set 4, $P = 1.62 \times 10^{-6}$; Set 5, $P = 2.43 \times 10^{-6}$; p-values were corrected for multiple hypothesis testing by the Benjamini-Hochberg method), demonstrating that the observed enrichment is robust to the choice of negative controls.

(C) Density plot comparing the average Hi-C connectivity scores of interacting AP/MS C2H2-ZFP pairs (94 pairs) with negative control pairs (3,476 pairs). Interacting AP/MS pairs exhibited significantly higher connectivity scores than negative controls (Mann–Whitney test).

(D–F) Density plots comparing Hi-C connectivity scores for subsets of interacting LUMIER C2H2-ZFP pairs containing at least one KRAB-containing protein (D), no KRAB-containing proteins (E), or two KRAB-containing proteins (F), against the same negative control dataset. In each case, interacting pairs exhibited significantly higher connectivity scores than negative controls (Mann–Whitney test).

Supplemental Figure S8

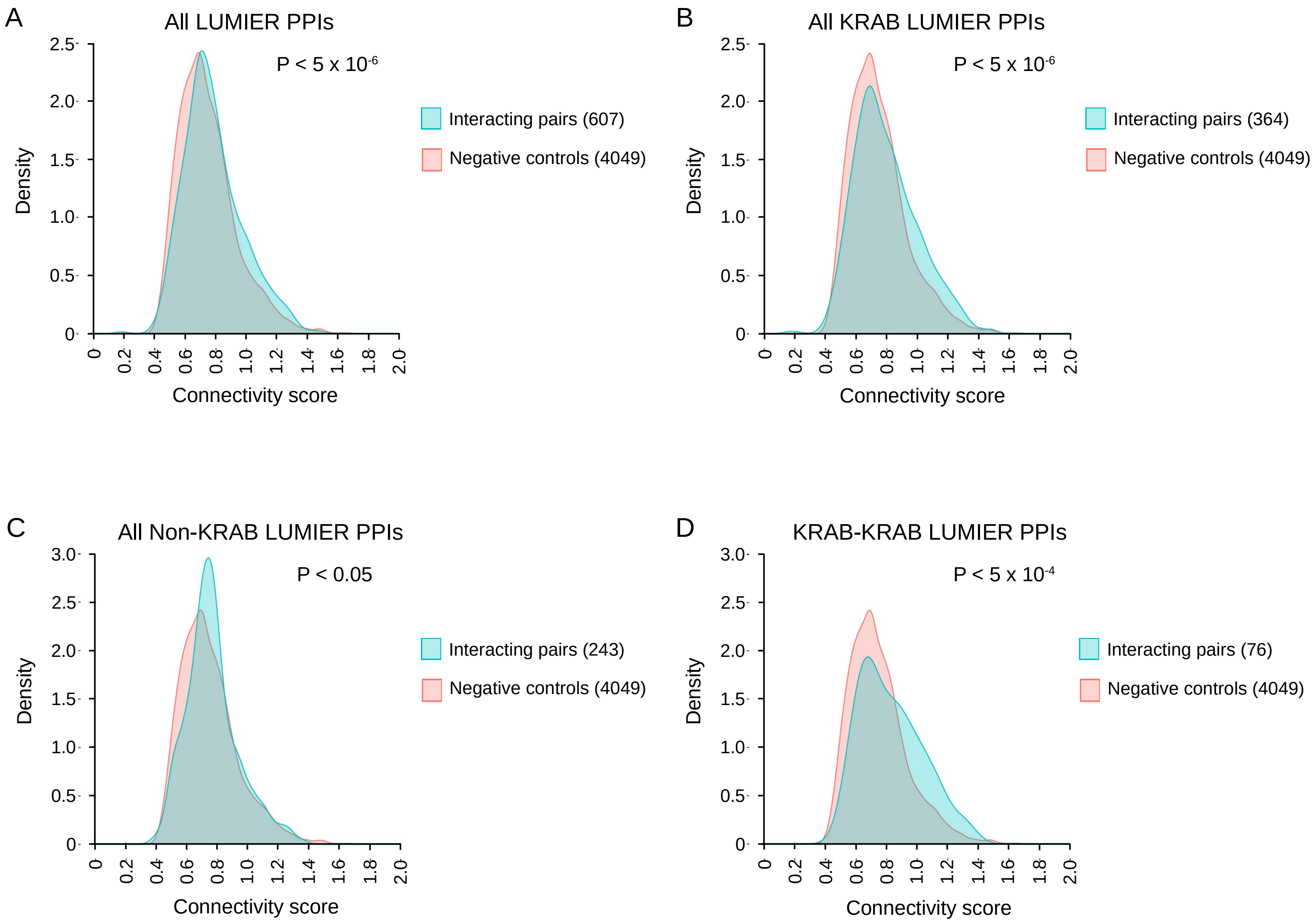

Supplemental Figure S8: Interacting KRAB-containing C2H2-ZFP pairs preferentially associate with Hi-C chromatin interactions outside RNAP II-associated chromatin.

Density plots comparing the average Hi-C connectivity scores of interacting LUMIER C2H2-ZFP pairs with negative control pairs after excluding chromatin interactions overlapping RNAP II ChIA-PET anchors.

(A) All interacting LUMIER C2H2-ZFP pairs.

(B) Interacting pairs containing at least one KRAB-containing C2H2-ZFP.

(C) Interacting pairs lacking KRAB-containing C2H2-ZFPs.

(D) Interacting pairs in which both proteins contain KRAB domains.

Supplemental Figure S9

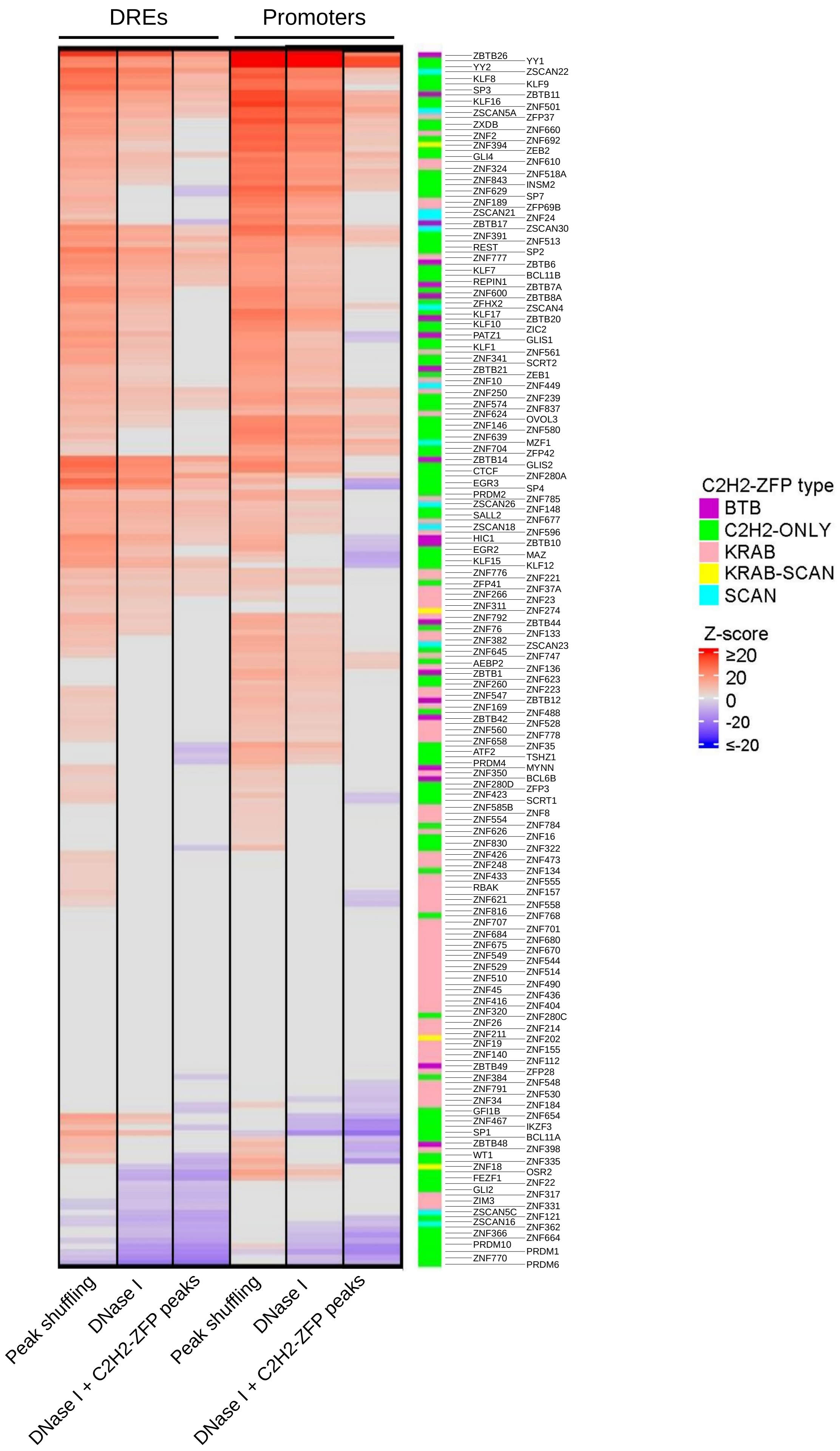

Supplemental Figure S9: C2H2-ZFPs DNA binding sites at LRI anchors are mutated in cancer. Heatmap showing Z-scores for the overlap of DNA-binding of C2H2-ZFPs with cancer mutations across DRE and promoter LRI anchors. The overlaps were done using three different null backgrounds for the C2H2-ZFP ChIP-seq datasets, which consisted of 1) a random chromosome-matched set of locations for the C2H2-ZFP ChIP-seq peaks within LRI anchors (Peak shuffling), 2) HEK293 DNase I hypersensitive sites within LRI anchors (DNase I), and 3) HEK293 DNase I hypersensitive sites along with the ChIP-seq peaks of all 216 C2H2-ZFPs analyzed in this study within LRI anchors (DNase I + C2H2-ZFP peaks). Only C2H2-ZFPs with significant Z-scores are displayed. Note that this figure is the enlarged version, with gene names indicated, of the main text Figure 5A.

Supplemental Figure S10

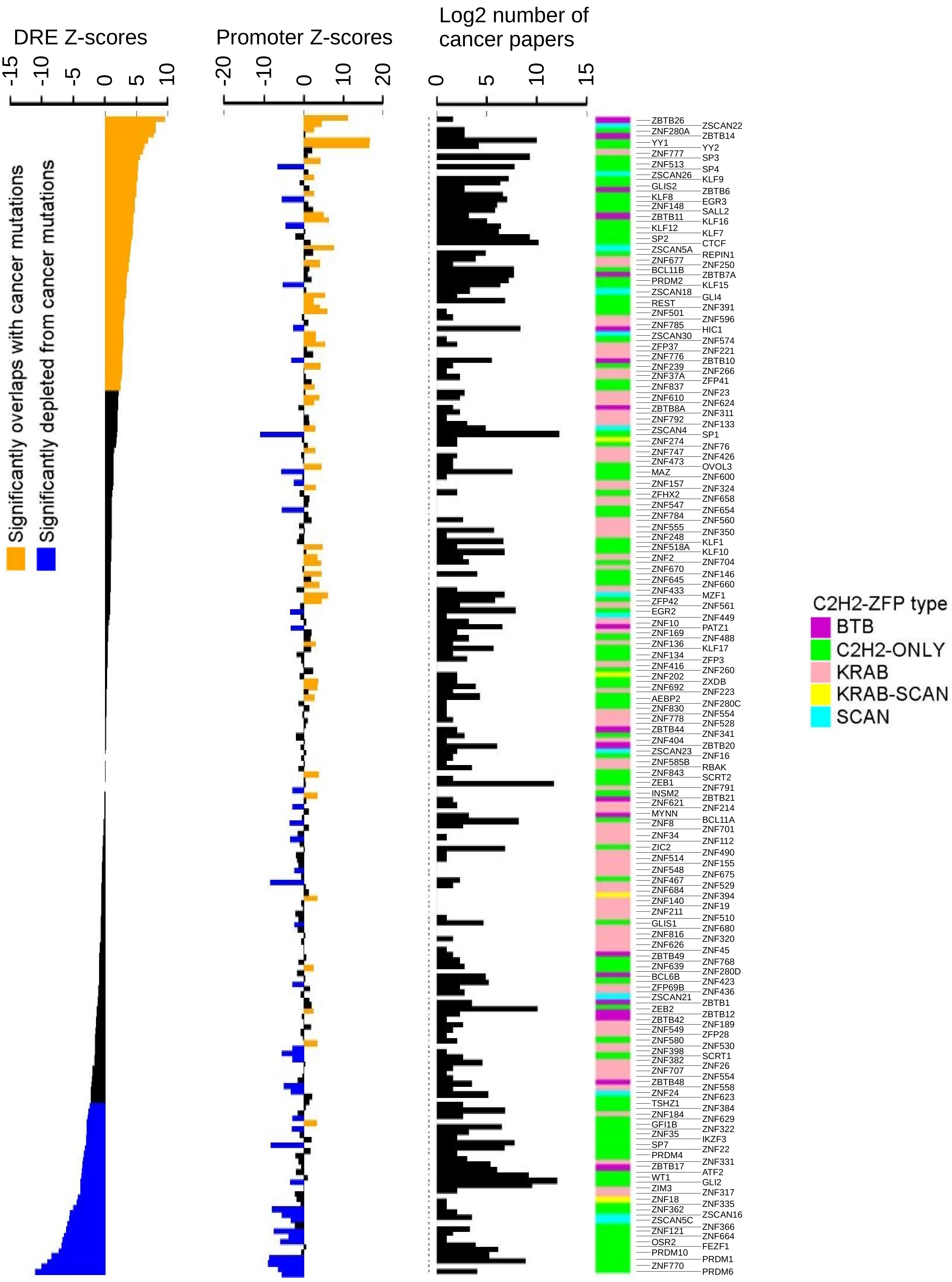

Supplemental Figure S10: DNA binding sites of C2H2-ZFPs at LRI anchors are mutated in cancer. Barplots displaying Z-scores for the overlap of the DNA-binding of C2H2-ZFPs with cancer mutations across DRE and promoter LRI anchors, as well as the number of papers that have been published for the C2H2-ZFPs related to cancer. The displayed Z-scores are the ones that were calculated using the DNase I + C2H2-ZFP peaks background in panel A. Note that this figure is the enlarged version, with gene names indicated, of the main text Figure 5B.

Supplemental Figure S11

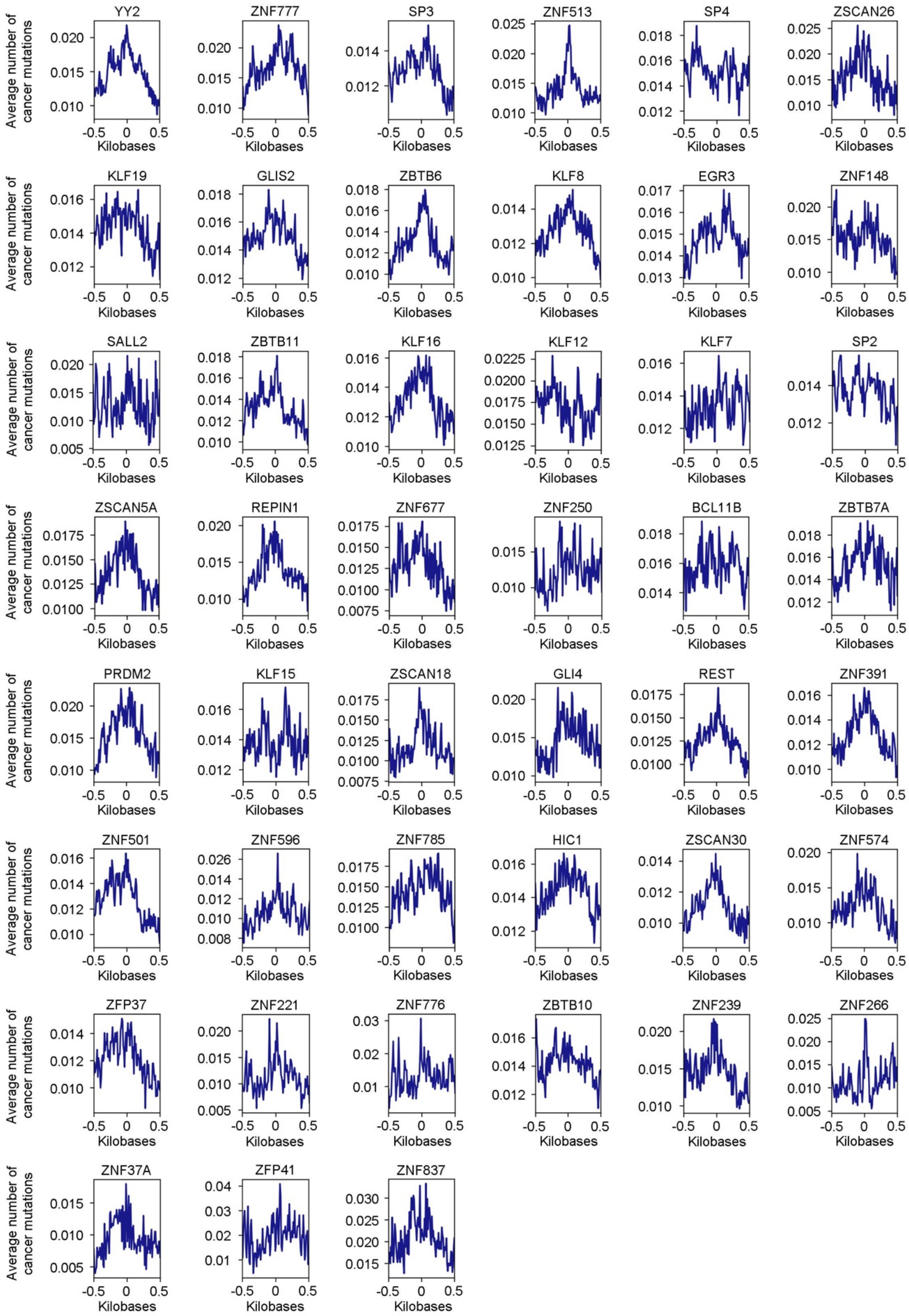

Supplemental Figure S11. Metaplot representation of cancer mutations around DNA-binding sites for C2H2-ZFPs at DRE LRI anchors +/- 500 bp. The figure only displays C2H2-ZFPs that overlap significantly with cancer mutations with all three different backgrounds used in Fig. 5A and that were not included in Fig. 5C.

Supplemental Figure S12

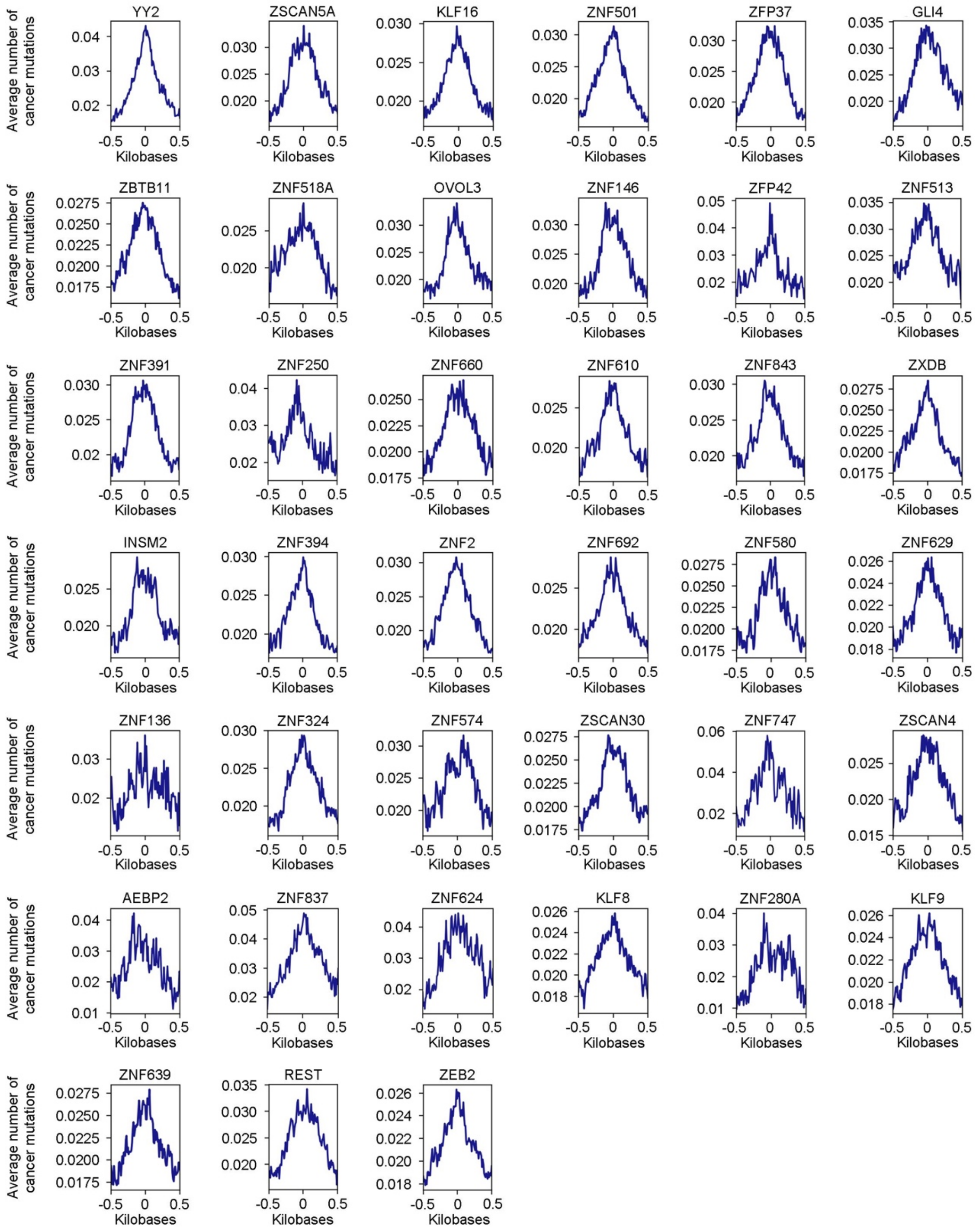

Supplemental Figure S12. Metaplot representation of cancer mutations around DNA-binding sites for C2H2-ZFPs at promoter LRI anchors +/- 500 bp. The figure only displays C2H2-ZFPs that overlap significantly with cancer mutations with all three different backgrounds used in Fig. 5A and that were not included in Fig. 5D.

Supplemental Figure S13

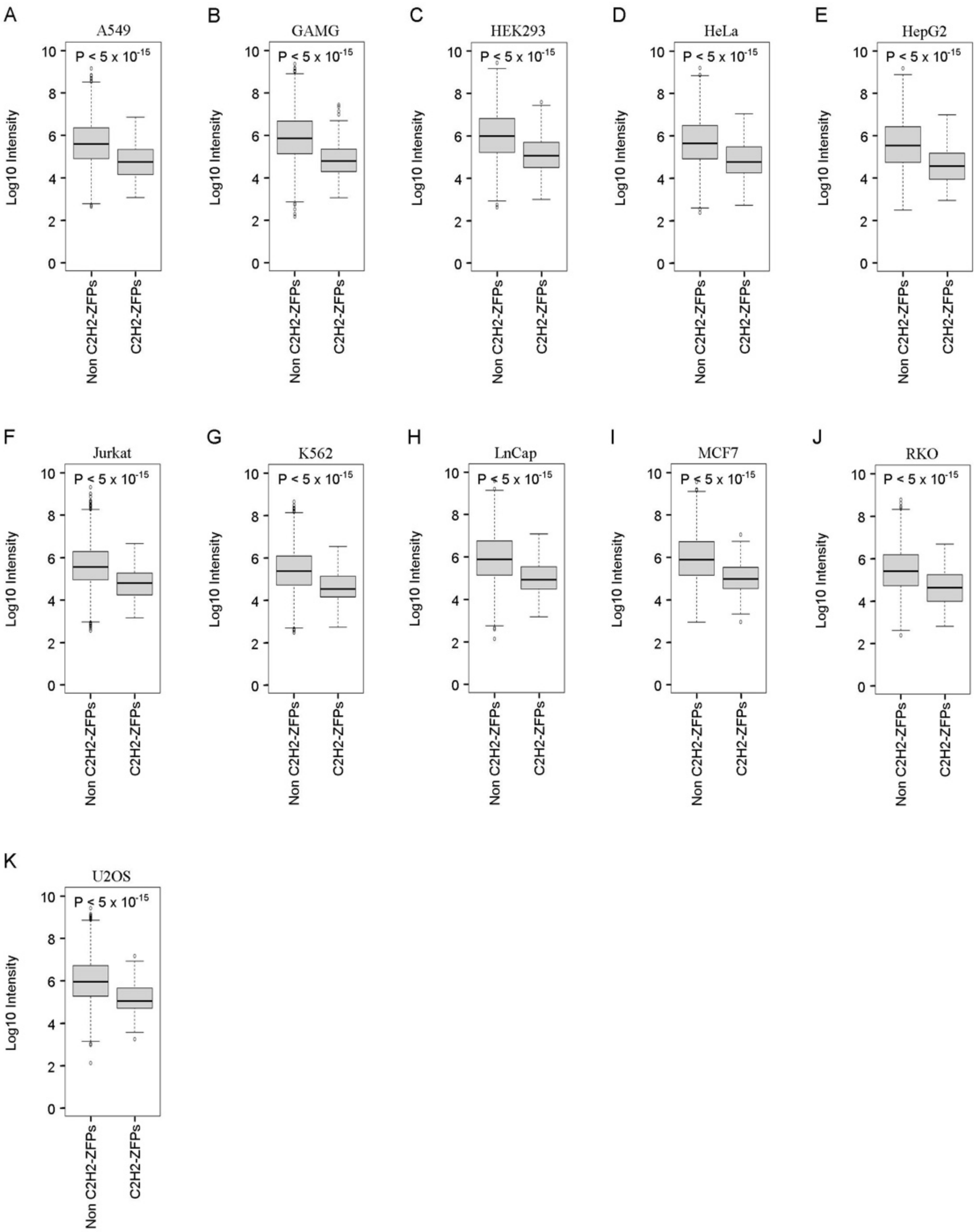

Supplemental Figure S13. C2H2-ZFPs are expressed at lower levels compared to non-C2H2-ZFP proteins in the human proteome.

(A-K) Boxplots comparing the expression of C2H2-ZFPs to non-C2H2-ZFP proteins in (A) A549, (B) GAMG, (C) HEK293, (D) HeLa, (E) HepG2, (F) Jurkat, (G) K562, (H) LnCap, (I) MCF7, (J) RKO, and (K) U2OS cell lines. The Log10 intensity scores after iBAQ normalization are used as a quantification of protein expression. The p-values were calculated by using the Mann-Whitney test.

Supplemental Figure 14

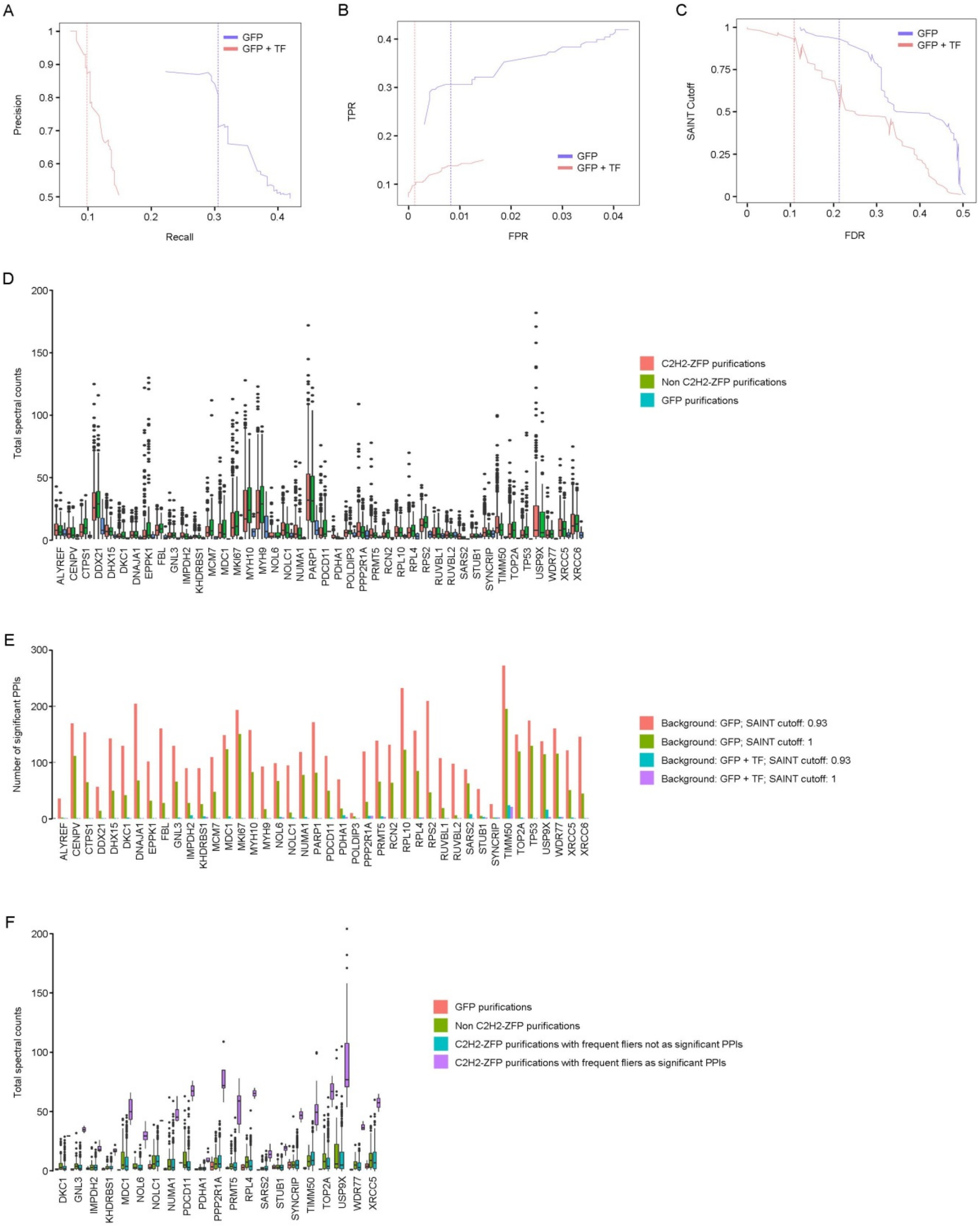

Supplemental Figure S14. Assessment of scoring method for C2H2-ZFP PPIs.

(A) Precision-recall curves comparing SAINT analyses using GFP only purifications (GFP) or GFP and non-C2H2-ZFP TFs as background (GFP + TF). Vertical lines indicate a SAINT cutoff of 0.93 for the GFP and GFP + TF curves.

(B) ROC curves comparing SAINT analyses using GFP only purifications or GFP + TF as background. Vertical lines indicate SAINT cutoff of 0.93 for the GFP and GFP + TF curves.

(C) Curves comparing FDR at different SAINT cutoffs using GFP only purifications, or GFP + TF as background. Vertical lines indicate SAINT cutoff of 0.93 for the GFP and GFP + TF curves.

(D) Boxplots showing the distributions of total spectral counts of some proteins that appear frequently in AP/MS experiments in GFP purifications, C2H2-ZFP purifications, and non-C2H2-ZFP TF purifications.

(E) Bargraph showing the number of significant PPIs involving some proteins that appear frequently in AP/MS experiments after SAINT scoring with GFP purifications or GFP + TF as background at SAINT cutoff of 0.93 and 1.

(F) Boxplots comparing the distributions of total spectral counts for proteins that appear frequently in AP/MS experiments that significantly interact with a subset of C2H2-ZFPs (SAINT score $\geq$ 0.93) to purifications done for GFP, non C2H2-ZFP TFs and C2H2-ZFPs that do not interact significantly with those proteins.
