# Supplementary material for: Integrative analysis reveals extensive interactions among C2H2 zinc finger proteins at chromatin loop anchors
Source: Nucleic Acids Res. 2026 Oct 19;54(19):gkag1004. doi: 10.1093/nar/gkag1004 (PMC13678222; doi:10.1093/nar/gkag1004)
Supplement: Supplementary file 1 [file gkag1004_supplemental_files.zip › Supplementary Files.docx]

**Supplemental Tables**

**Supplemental Table S1.** Resources used in the paper.

**Supplemental Table S2.** Statistics generated for the overlap of the C2H2-ZFP DNA-binding sites with LRI anchors.

**Supplemental Table S3.** Source of C2H2-ZFP constructs that were used in the paper.

**Supplemental Table S4.** SAINT scored PPIs of C2H2-ZFPs that were identified by AP-MS.

**Supplemental Table S5.** Statistics for analyses performed with DAVID on prey proteins of C2H2-ZFPs.

**Supplemental Table S6.** Table indicating the C2H2-ZFP interactors that belong to significantly enriched functional clusters based on analysis with DAVID (59). The prey proteins from the clusters that have been highlighted in green in Supplemental Table S5A are being displayed in this table. Only prey proteins associated with terms that were significantly enriched in each cluster after multiple hypothesis correction are being displayed in the table (p-value < 0.05, Benjamini corrected).

**Supplemental Table S7.** GO terms and KEGG pathways that were significantly enriched for prey proteins of C2H2-ZFPs from different subfamilies.

**Supplemental Table S8.** PPIs among C2H2-ZFPs that were identified by AP-MS.

**Supplemental Table S9.** PPIs among C2H2-ZFPs that were identified by LUMIER.

**Supplemental Table S10.** DOVC and DOVT values for interacting C2H2-ZFP pairs.

**Supplemental Table S11.** Statistics generated for the overlap of the C2H2-ZFP DNA-binding sites with cancer mutations at LRI anchors.

**Supplemental Table S12.** p-values for analyses done in Supplemental Fig. S6.

**Supplemental Table S13.** Classification of C2H2-ZFPs into age groups.

**Supplemental Data**

**Supplemental Data S1**

Files providing an interactive network for Fig. 3A, C. An interactive network session can be initiated by unzipping each folder and clicking on the icons labelled as “index”.

**Supplemental Data S2**

Tables providing raw data for each prey protein that was analyzed by LUMIER. The name of the prey protein is indicated in the file name. In each file, column 1 lists the LUMIER intensity signal, column 2 lists the 96 well plate in which the interaction of the bait and prey protein was tested, column 3 lists the plate well in which the interaction of the bait and prey protein was tested, column 4 lists the name of the prey construct used in the LUMIER test.
